# Healthcare system readiness to manage viral hepatitis in Viet Nam and the Philippines: results of a brief health facility assessment

**DOI:** 10.1186/s12913-026-14088-y

**Published:** 2026-02-02

**Authors:** Martin Louis Fernandez, Hoang Nguyen, Dang Nguyen, Bethany Holt, Duong Doan, Manu Gaspar, Geohari Hamoy, Jhaki Mendoza, Timothy Bill Mercado, Daniel Joy Cabauatan, Huyen Nguyen, My Dang, Vy Nguyen, Janus Ong, Joseph Michael Manlutac, Yen Nguyen, Hoa Nguyen, Dung Vu, Jan Philip Florendo, Danica Delima, Mary Cris Rombaoa, Jose Mateo Dela Cruz, Rosanna Buccahan, Hjordis Marushka Celis, Jeanette Lazatin, Pham Nam Thai, Pham Xuan Truong, Tran Khanh Thu, Thuy Pham, David Duong, Todd Pollack

**Affiliations:** 1 National Institutes for Health, University of the Philippines, Manila, Philippines; 2 The Partnership for Health Advancement in Viet Nam (HAIVN), Hanoi, Viet Nam; 3 Program in Global Primary Health Care, Office for Research Initiatives and Global Programs, Harvard Medical School, Boston, MA USA; 4 Division of Global Health Equity, Brigham and Women’s Hospital, Boston, MA USA; 5 College of Health Sciences, VinUniversity, Hanoi, Viet Nam; 6 Action to the Community Development Institute, Hanoi, Viet Nam; 7 Menzies Institute for Medical Research, University of Tasmania, Tasmania, Australia; 8 Thai Binh University of Medicine and Pharmacy, Thai Binh, Viet Nam; 9 School of Health Sciences, University of the Philippines Manila, Tarlac, Philippines; 10 Thai Binh Department of Health, Thai Binh, Viet Nam; 11 Department of Medicine, Beth Israel Deaconess Medical Center, Boston, MA USA; 12 Department of Health, Central Luzon, Pampanga, Philippines; 13 Tarlac Provincial Hospital, Tarlac, Philippines; 14 Provincial Health Office, Province of Bataan, Bataan, Philippines; 15 Provincial Health Office, Province of Bulacan, Bulacan, Philippines; 16 Provincial Health Office, Province of Tarlac, Tarlac, Philippines; 17 Graduate School of Nursing and Public Health, Angeles University Foundation, Angeles City, Philippines; 18 Harvard T.H. Chan School of Public Health, Harvard University, Boston, MA USA; 19 Department of Social Medicine, College of Medicine, University of the Philippines, Manila, Philippines

**Keywords:** Viral hepatitis, Health facility assessment, Healthcare readiness, Primary healthcare, Philippines, Viet Nam

## Abstract

**Background:**

Chronic hepatitis B (HBV) and hepatitis C (HCV) pose significant public health challenges in Viet Nam and the Philippines. Both countries have initiated national strategies aimed at decentralizing hepatitis care to primary healthcare facilities, but the availability and readiness of these facilities to manage hepatitis remains unclear. This study aimed to evaluate the availability and readiness of primary care facilities in Viet Nam and the Philippines to provide comprehensive hepatitis services and to identify key gaps in care delivery.

**Methods:**

A mixed-methods approach was used, combining health facility surveys and focus group discussions (FGDs) with healthcare workers to assess service availability and identify barriers and enablers of hepatitis care. Data were collected from 18 health facilities and 36 healthcare workers through 6 FGDs across both countries.

**Results:**

The study identified critical gaps in hepatitis service availability, healthcare worker training, diagnostic capacities, and community engagement at the primary care level. While provincial level facilities were well-equipped, most primary care facilities lacked essential resources such as viral load testing, medications, and adequately trained healthcare workers. A key barrier was the lack of social health insurance coverage or reimbursement for hepatitis services at the primary care level in both countries.

**Conclusion:**

Gaps identified may require coordinated action from both national and subnational stakeholders. Expanding social health insurance coverage to include hepatitis services at the primary care level, improving healthcare worker training and support, and ensuring the availability of diagnostics and antivirals at the primary care level are essential steps to meet the 2030 hepatitis elimination targets in Viet Nam and the Philippines.

**Supplementary information:**

The online version contains supplementary material available at 10.1186/s12913-026-14088-y.

## Introduction

Viral hepatitis is a major public health concern, with an estimated 296 million people living with chronic hepatitis B virus (HBV) and 58 million with chronic hepatitis C virus (HCV) worldwide [1, 2]. According to the World Health Organization (WHO) data from 2022, only 13% of all people estimated to be living with HBV were aware of their infection, and only 3% of those diagnosed were on treatment [3]. Among persons living with HCV, an estimated 36% knew their diagnosis and only 20% of those diagnosed had been treated with direct acting antivirals (DAAs) by the end of the year [4]. Untreated chronic infection may lead to liver cirrhosis and liver cancer, which together account for 3.5% of all deaths worldwide [5]. In 2020, Asian countries represented 73.3% of global deaths from liver cancer. Liver cancer was the fifth most common cancer in Asia and HBV and HCV infections remain the most common risk factors [6]. In 2022, the WHO endorsed an updated, integrated global health sector strategy for addressing Human Immunodeficiency Virus (HIV), viral hepatitis, and sexually transmitted infections (STIs) with a focus on people-centered, decentralized and simplified service delivery models for HBV and HCV [7].

The Philippines and Viet Nam are both ranked among the highest in the Western Pacific region and the top 20 globally in the burden of hepatitis. In Viet Nam, the prevalence of HBV is estimated at 6.0%, while HCV seroprevalence is less than 1.0% [8]. In the Philippines, the prevalence of HBV is estimated to be 4.9% and HCV prevalence is lower at 0.6% [9–11]. Among people who inject drugs (PWID) in Viet Nam, the HCV prevalence ranges between 31.0% and 97.2%, but only a third of PWID in Hanoi have ever been tested for HCV, highlighting a significant gap in access and/or uptake [12]. To address this high burden, especially among specific populations, both countries have implemented national programs for viral hepatitis prevention and control and are committed to achieving the WHO viral hepatitis elimination goals [13–15]. Viet Nam released its second national hepatitis strategy in 2021 and since then has received support from the Global Fund to Fight AIDS, Tuberculosis, and Malaria to the Ministry of Health (MOH) to deliver HCV treatment for people living with HIV (PLHIV) and patients on methadone maintenance treatment [16]. The Philippines, on the other hand, incorporated hepatitis elimination into its national strategy on HIV, AIDs, and STIs Prevention and Control in 2017, led by a national Technical Working Group. Since 2019, the Department of Health (DOH) has supported a sub-national hepatitis initiative for integrated service delivery of HBV and HCV care through a network of HIV facilities in Central Luzon; however, the initiative’s expansion was halted due to the COVID-19 pandemic [17].

A key component of the viral hepatitis elimination strategy in both countries is the decentralization of hepatitis care to the primary healthcare level. A recent policy analysis found that both countries have relatively robust policy frameworks in place to support this approach [9–11]. However, data is limited on the capacity of primary care facilities in Viet Nam and the Philippines to deliver hepatitis services in line with those policies [9, 18–21]. We therefore aimed to determine the readiness of primary healthcare facilities in both countries to deliver comprehensive services for the management of chronic viral hepatitis and to identify key gaps in care delivery. A health facility assessment (HFA) is a tool to assess the availability of health services and the capacities of health facilities to provide services at required standards of quality [22]. By periodically identifying the gaps in the primary care system, HFAs enable policy-makers, program managers, and healthcare providers to prioritize investments, allocate resources strategically, and develop targeted interventions to improve the quality and accessibility of health services [23].

## Methods

### Research design

The study applied a mixed methods of data collection including two components: (1) a health facility survey on service availability and readiness and (2) focus group discussions (FGDs) with health providers focusing on identifying the enablers and barriers to hepatitis care service provision.

### Research setting

The study was conducted in late 2022 up to early 2023 in Thai Binh province in Viet Nam and in Central Luzon (Region III) in the Philippines. Within each locality, nine health facilities were purposely selected to capture multiple levels of the health system required to provide integrated care for HBV and HCV (see Table 1). Selection of specific facilities was based on recommendations from provincial health authorities, who were asked to identify facilities that would provide geographic representation across each province. While facility size, patient volume, and service scope were not used as formal criteria, the intention was to include a mix of urban and rural settings, ensuring representation from different catchment areas and distances from referral hospitals. In Thai Binh, facility selection included sites from districts located near, intermediate, and far from the provincial hospital. In Central Luzon, one provincial-level facility and two primary care-level facilities were selected per province (Bataan, Bulacan, and Tarlac) to reflect variation in local health system organization and geography.

**Table 1:** Viet Nam and Philippines healthcare facilities involved in the study

Health system level	Viet Nam	The Philippines
Provincial level	**Provincial level facilities** - One provincial general hospital	**Provincial level facilities** - Three level 3 hospitals (one from each of 3 provinces in Region III included)
Primary care level	**District level facilities** - Two district health centers/hospitals **Commune level facilities** - Six commune health stations	**District level facilities** - Two district care facilities (custodial care facility and level 1 hospital) **Community level facilities** - Four community care facilities (including rural health unit, social hygiene clinic, and primary HIV care clinic)

In Viet Nam, the primary care level includes commune health stations, district hospitals and health centers. In the Philippines, the primary care level includes community care facilities (i.e., rural health units, social hygiene clinics, and HIV care clinics), as well as district care facilities (i.e., custodial care facilities and level 1 hospitals). In both countries, these primary care level facilities serve as the first point of contact for most of the population.

#### Central Luzon, Philippines

The Philippines’ healthcare system is a mixed public-private structure, which was significantly decentralized by the Local Government Code of 1991. In 2021, the Mandanas-Garcia Ruling increased local government accountability and authority for healthcare, transferring more functions (including greater responsibility for funding and procurement of diagnostics) to local government units. [9] This study included three of the seven provinces in Region III (Central Luzon), specifically Bataan, Bulacan, and Tarlac, chosen for their parts in the DOH’s Hepatitis Demonstration Project. These provinces cover approximately 7203 square kilometers with a combined population of around 6.07 million. Out of 175 public health facilities in the three provinces, three provincial level hospitals, two district level facilities, and four community care facilities were selected to participate in the study. Provincial level hospitals provide both inpatient and outpatient services, including general and specialist care; district level facilities provide general inpatient and outpatient services; and community care facilities offer outpatient services only [17, 24–29].

#### Thai Binh, Viet Nam

Viet Nam’s public healthcare system is organized in a pyramid framework with four administrative levels: central, provincial, district, and commune. Central-level hospitals, at the apex, provide specialist services and technical support and training for the lower levels [30]. Thai Binh province, located in the Red River Delta in northern Viet Nam, spans 1,585 square kilometers and includes one city and seven districts, with an average population of 1.9 million. There are 21 public health facilities at the provincial level (nine hospitals and 12 preventive medicine centers), 12 district hospitals, eight district health centers, and 260 commune health stations. Five private hospitals also provide healthcare services in Thai Binh. The provincial and district hospitals deliver clinical care and treatment services, whereas district health centers and commune health stations traditionally have focused on preventive care and public health services [31].

### Research instrument development

A health facility assessment survey, adapted from the WHO Service Availability and Readiness Assessment (SARA) tool, was developed to measure specific service availability in each health facility [32]. This survey included the type of facility, healthcare worker capacity and capability, and the presence of other hepatitis-related services across the continuum of care from prevention to treatment and monitoring, aligned with national guidelines and policies. Services assessed included prevention services (such as information, education, communication (IEC) activities and vaccination), diagnostic services (screening and confirmatory testing), and treatment and monitoring services (medication supplies, healthcare worker skills and capacities). The survey was adapted for local guidelines, language, and context in each country.

A focused group discussion (FGD) guide was developed to gather information from healthcare facility representatives, including frontline workers and leaders, where the health facility assessment surveys were conducted. The guide consisted of open-ended questions to elicit participants’ general knowledge about HBV and HCV and their perceptions of barriers and enablers in providing hepatitis-related care. The questionnaire was initially developed in English and translated into Filipino and Vietnamese, with back-translation ensuring content consistency.

### Data collection

Data was collected through a three-step process from late 2022 to early 2023 including distribution of survey forms to facilities, site visits to verify survey data, and FGDs with healthcare providers.

#### Health facility survey

Following consent to participate in the study, surveys were distributed via electronic mail to the 18 health facilities in both the Philippines and Viet Nam. The survey was distributed to a designated point person at each facility, typically identified by the facility leadership. This individual was responsible for coordinating completion of the survey and facilitating input from relevant staff members, including nurses, physicians, and medical technologists, depending on the facility’s staffing structure. This collaborative approach was intended to ensure that responses reflected a comprehensive and accurate picture of service availability and readiness. Each facility was given a month to complete and return the completed survey. Survey responses were then validated during one to two-hour face-to-face site visits by study team representatives.

#### Focus group discussion

Six FGD sessions were conducted: four in the Philippines and two in Viet Nam, with four to eight participants per session. The FGD participants include representatives of the administrative and clinical departments of the healthcare facilities. The discussions explored providers’ views on the enablers and barriers within the current systems for caring for people with hepatitis. Each session lasted for about one hour and was facilitated by two local study staff from the research team. While one worked as a facilitator and asked questions, the other observed and documented key messages into categories of barriers or enablers. Sessions were audio recorded with participant consent and the data were subsequently transcribed and translated into English. Following the session, study staff listened to the audio recording to ensure accuracy.

### Data analysis

#### Quantitative

Quantitative data were analyzed using descriptive statistics. Data from the health facility surveys were compiled in Google Sheets, accessible only to the study team members from each respective country. The availability of hepatitis-related clinical services was mapped across the continuum of care to identify gaps in service availability. The data were compared across different levels and types of health facilities.

#### Qualitative

**Fig. 1:**
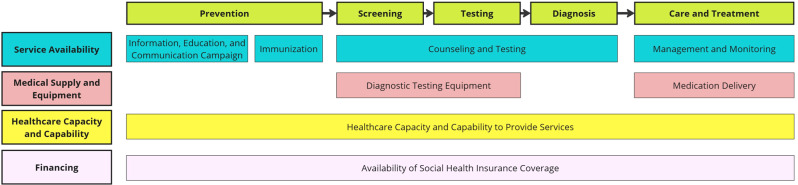
Conceptual framework for health facility assessment and analysis

FGD data were analyzed by adapting a rapid deductive qualitative analysis (RDQA) approach [33], adapted from a methodological study validating it as an alternative to traditional methods. The analysis was guided by a conceptual framework (Fig. 1) developed from the WHO’s SARA framework and the continuum of care for chronic viral hepatitis. Prior to the FGDs, a coding matrix was developed in Microsoft Excel® to capture the enablers and barriers to hepatitis-related care across the patient journey.

Hepatitis healthcare services were identified and categorized according to themes and were subsequently correlated with findings from the health facility assessment survey. The themes were identified after the FGDs based on commonly occurring topics and alignment with the conceptual framework. Based on the data, key results were identified through consensus among research teams and validated in participatory workshops in each country, determining convergences and country-specific themes.

## Results

### Response rate and participant demographics

All 18 of the health facilities invited to participate in the study completed the health facility survey. For the FGDs, in the Philippines, eight of the nine facilities (88.89%) were represented in the discussion, while in Viet Nam, all nine facilities (100%) were represented. The demographics of the participants based on profession, gender, and facility type per country are shown in Table 2.

**Table 2:** Basic demographics of the 18 health workers who participated in the focus group discussions in each country

Categories		Philippines (n = 18)	Viet Nam (n = 18)
Health Facility Level	Provincial	8 (44.4%)	2 (11.1%)
	Primary Care	10 (55.6%)	16 (88.9%)
Profession	Physician	4 (22.2%)	12 (66.7%)
	Nurse	13 (72.2%)	4 (22.2%)
	Medical Technologist	1 (5.6)	2 (11.1%)
Gender	Male	8 (44.4%)	10 (55.6%)
	Female	10 (55.6%)	8 (44.4%)

### Quantitative data

The availability of hepatitis-related services is described below and summarized in Tables 3 and 4.

**Table 3:**
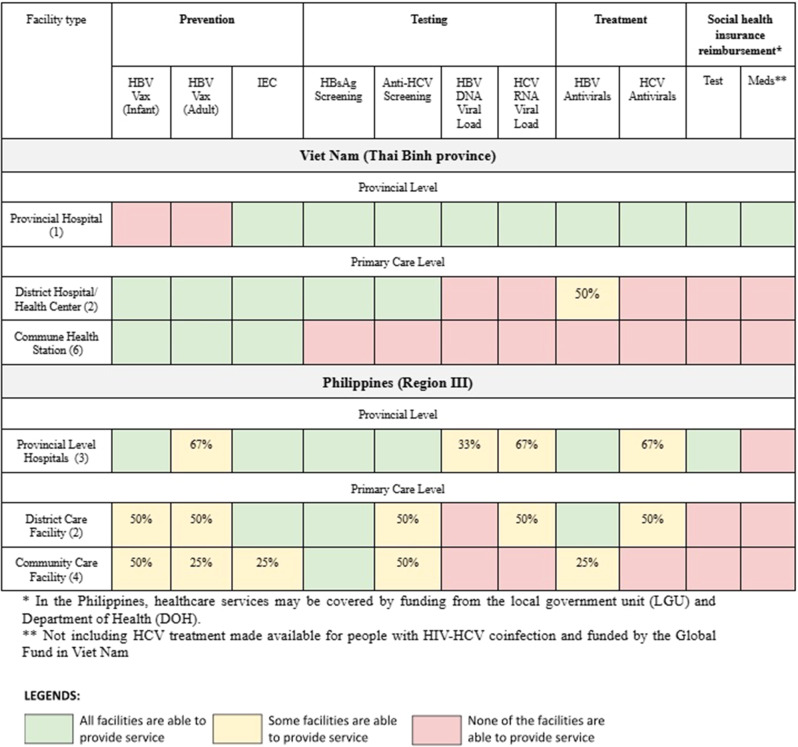
Availability of the continuum of services and social health insurance coverage for hepatitis B and C among surveyed facilities in Viet Nam (Thai Binh province) and the Philippines (Bataan, Bulacan, and Tarlac)

**Table 4:**
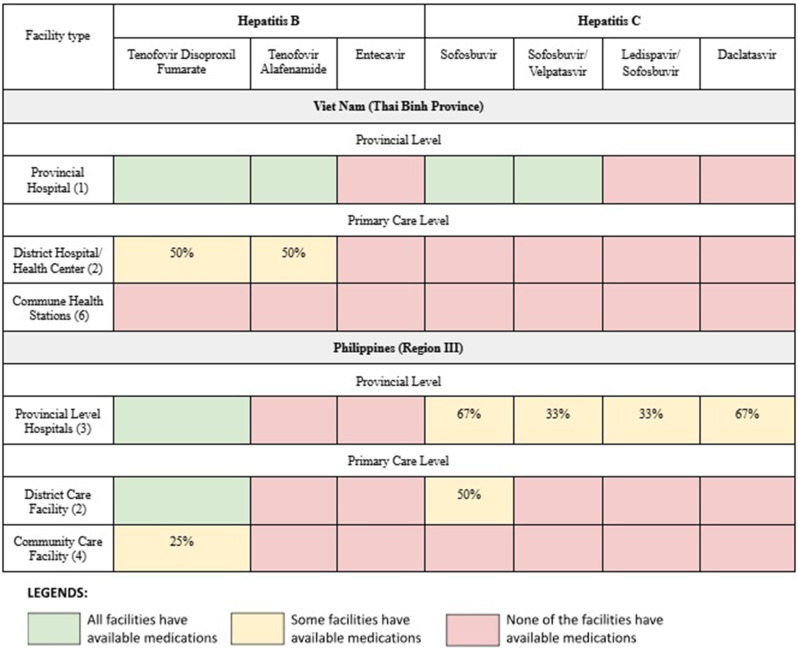
Available antiviral medications for treatment of chronic HBV and HCV infection in Viet Nam and the Philippines

#### Prevention

In the Philippines, prevention services through HBV vaccination and IEC activities were available at all levels of the system. However, infant vaccination services were more available (6 out of 9 facilities or 67%) compared to adult vaccination services (4 out of 9 facilities or 44%). IEC activities were being implemented at all the provincial and district level facilities but only one out of four community care facilities. In Viet Nam, IEC activities and HBV vaccination were available at all surveyed district and community level facilities. The Thai Binh provincial hospital conducted IEC activities but did not provide hepatitis vaccination services.

#### Testing

All facilities surveyed in the Philippines were able to conduct screening for hepatitis B surface antigen (HBsAg) however their capability differed in terms of HCV antibody screening and both HBV and HCV viral load testing. HCV antibody screening was available at all provincial level facilities and half of the district level and community care facilities. Only one out of the nine (11%) health facilities surveyed were able to conduct HBV DNA testing in-facility, while two of three provincial level facilities and one of the two district level facilities were able to conduct HCV RNA testing. In Viet Nam, screening for HBsAg and anti-HCV were available at both provincial and district hospitals. However, viral load testing services were only accessible at the provincial level.

#### Treatment

In the Philippines, the availability of medications for treatment of chronic HBV and HCV varied among the health facilities. In general, HBV antivirals were more readily available than HCV antivirals. Most provincial and district level facilities had HBV treatment available, while only one community care facility was able to provide treatment for HBV and no community care facility could provide treatment for HCV. Tenofovir Disoproxil Fumarate (TDF) was available in six out of nine health facilities, but only one of four community care facilities. HCV medications were primarily available in provincial level facilities; however one district level facility had sofosbuvir available. Among community care facilities, HBV medications were limited to one facility and none had HCV medications. In Viet Nam, only the provincial hospital could provide medications for both HBV (TDF and Tenofovir Alafenamide [TAF]) and HCV (Sofosbuvir/Velpatasvir). One district hospital could dispense TDF with technical assistance from the provincial hospital. No commune health stations had medications for either HBV or HCV.

#### Health insurance coverage

No primary care facilities surveyed in either country were able to provide hepatitis services under the national social health insurance system. In the Philippines, some coverage was available through the provincial level, but only for in-patient testing services, whereas in Viet Nam, only the provincial level could provide hepatitis testing and medication services under the national social health insurance system.

### Qualitative data

A total of 36 health workers from 18 health facilities underwent FGDs using the semi-structured discussion guide. Identified themes included community awareness and education, active screening opportunities, healthcare worker capacity, diagnostic and medication supply, affordability, and linkage to care. The results are presented by theme and barriers and facilitators are discussed within each identified theme.

#### (1) Community awareness and IEC services

Healthcare workers in both countries highlighted insufficient community awareness as a major barrier to hepatitis care. Participants felt that a lack of health literacy around viral hepatitis has led to challenges in encouraging patients to screen and link to care, even when testing supplies or treatment are available.

*Patients are not well-informed on what hepatitis is and what should be done, even if a healthcare worker explains it. - nurse, the Philippines*

*The IEC activities related to hepatitis prevention and treatment services are being conducted but are still inadequate at the community level. - nurse, Viet Nam*

*It’s very difficult to conduct intervention on pregnant women … We, doctors, counsel them but cannot convince them to take medicine as the disease went silently for decades. - doctor, Viet Nam*

#### (2) Active screening opportunities

Health workers in both countries reported that hepatitis screening was being conducted for specific populations. For example, HBV screening is being integrated into blood donation drives, prenatal consultations, and pre-employment health assessments (particularly relevant for military service in Viet Nam). Additionally, patients undergoing hemodialysis are offered screening for HCV. Participants believed that such efforts enhance the diagnosis and prevention of HBV and HCV within these specific subgroups.

*“Active hepatitis B screening service is offered by various types in Viet Nam, including blood donation drives, prenatal consultations, and pre-employment health assessments.” - doctor, Viet Nam*

*“For us, we have routine screening of pregnant women. There is also a routine screening of dialysis patients which is another key population for hepatitis. Since we are located in the HIV treatment hub, we also encourage [the patients to get tested] since we have available test kits for HIV and we also include Hepatitis as well. [Hepatitis] is also included in the screening of clients with multiple sex partners. “- nurse, the Philippines*

#### (3) Health worker capabilities and capacities

Healthcare workers in the Philippines described feeling confident in their ability to provide pre-test and post-test counseling services to patients. In survey sites in Viet Nam, counseling services are often applied in the surgery department at district hospitals where screening for hepatitis and other infectious diseases (such as HIV) are offered to reduce transmission risk to healthcare workers. Also, women in the late stages of pregnancy receive hepatitis counseling services as recommended by the MOH. However, these practices are still limited at the primary care level.

With the exception of a few individuals from one province in the Philippines, most healthcare providers in both countries expressed a significant lack of confidence in managing patients with hepatitis B and/or C. This lack of confidence was attributed by participants to insufficient training. In the Philippines, providers explained that this challenge is compounded by a scarcity of manpower, forcing them to juggle responsibilities across various health programs. This dispersion of focus and resources leads to gaps in management for these patients. In Viet Nam, many healthcare staff at district hospitals lack the counseling skills needed to persuade patients to undergo hepatitis B and C screening. In addition, health workers often do not have the certification required for providing hepatitis diagnosis and treatment under social health insurance as physicians are required to have disease-specific specialized training certificates to qualify for health insurance coverage

*“I think the question would be what is happening in the field as [the healthcare workers] are taking care of an abundant amount of patients with a wide variety of cases. This is what we are used to due to manpower issues and we do not have any choice, other than to accept [the situation] …” - nurse, Philippines*

*“As recommended by the MOH, we may offer screening for hepatitis and infectious diseases to the surgery patients or pregnant women as a prevention activity for both healthcare workers and patients. However, I don’t think it has been done widely in the hospitals at the district hospitals [primary care level]” - doctor, Viet Nam*

*“Same with other rural health units, we handle all of the [health] programs of the DOH. However, we still do not have training for Hepatitis B [management]. The medical technologist was the only one who was trained for hepatitis B. I think the training is really what is lacking in our facility.” - nurse, Philippines*

#### (4) Diagnostic and medication availability

Health workers in both countries reported a lack of diagnostics (screening and confirmatory test kits) and medications at the primary care level. Health workers felt that the inconsistent or lack of available commodities discouraged patients from utilizing the primary care level for hepatitis related services

*“… patients with hepatitis B are not monitored because our screening kits are limited, they are intended for pregnant people.” - nurse, the Philippine*

*“Our district hospital just has screening tests and drugs for hepatitis B treatment because of the small number of managed patients. Patient with hepatitis C get the treatment at higher levels such as provincial hospital or central hospitals” - doctor, Viet Nam*

#### (5) Affordability of care

Health workers from both countries expressed concern about the cost of hepatitis care and the barriers related to health insurance coverage for testing and treatment services. In Viet Nam, participants relayed that although hepatitis tests and medications are included in the national social health insurance package, many individuals still face challenges in accessing coverage and paying for care. In contrast, healthcare workers in the Philippines clarified that there is no general outpatient PhilHealth coverage for hepatitis, except for special populations including the indigent and HIV populations, nor a national health policy specifically for individuals with hepatitis, unlike the coverage available for HIV. The lack of health insurance coverage discourages health workers from actively promoting hepatitis testing or treatment.

*“Finance is a major barrier for patients. If the insurance pays a part, it will be much less burden for the patient, people will be more interested in treatment” – doctor, Viet Nam*

*“We do not promote [these hepatitis services] because these are still insufficient and … patients have to pay out of pocket for viral load testing and sometimes it is too expensive.” - nurse, the Philippines*

*“If we want to aim for sustainability, then the establishment of a health policy for hepatitis B should be there. The health policy could include the [hepatitis B] program within PhilHealth. [Since], if there is no health financing, the Rural Health Units do not generate income.” - nurse, the Philippines*

#### (6) Linkage to care practices

Health workers from both countries reported having no difficulties in referring patients to higher levels for treatment. As noted in one province within the Philippines, there is an efficient and effective communication channel between general practitioners and specialists. This streamlined communication facilitates timely patient referrals to specialists for appropriate management. Furthermore, specialists collaborate in co-managing patients when necessary. However, the back-referral process is not consistent and, as a result, primary care physicians often received no updates on their patients’ treatment plans.

*“Truth be told, actually, from the time of implementation, I didn’t see much of hindrances even on the part of the specialists. They’re accessible, and we’re available to refer as a primary care for them.” - doctor, the Philippines*

*“… The patients do not return to their respective [primary] health centers and their private OB’s are the ones managing them. So we do not have their records to know if they proceeded with management [for hepatitis].” - doctor, the Philippines*

*“We do not have any problem transferring the patients to higher levels based on their conditions … Some patients want to come back the district level for treatment but we cannot provide services because lack of drug and viral load tests for assessment, especially for hepatitis C” - nurse, Viet Nam*

## Discussion

The WHO recommends simplified primary care-based approaches to service delivery for both hepatitis B and C [34, 35]. This is supported, in part, by the HIV literature as well as a recent global systematic review and meta-analysis demonstrating that decentralization and integration of hepatitis C care to primary care improved access to testing, linkage, and treatment [34, 36]. However, decentralization of hepatitis services globally has been slow [37]. Our study demonstrated significant gaps in the readiness of primary healthcare facilities in Viet Nam and the Philippines to provide hepatitis related services.

One key finding from our analysis was the lack of healthcare workers with sufficient capacity in the management of viral hepatitis. In both countries, healthcare workers reported insufficient training in hepatitis management, which contributed to low confidence in providing care for people living with viral hepatitis. This finding is consistent with prior studies demonstrating a gap in the knowledge, attitude, and skills around viral hepatitis in the health workforce generally [38]. Enhancing healthcare worker capability through pre-service and in-service training, supported supervision and other capacity building strategies is an essential step to improve the readiness of primary care systems to manage viral hepatitis [39, 40].

Healthcare workers in both Viet Nam and the Philippines identified low community awareness as one barrier to early diagnosis and treatment. Despite existing IEC activities, general awareness remains insufficient, particularly in rural and underserved areas where misconceptions about hepatitis and stigma surrounding hepatitis persist [41, 42]. These findings underscore the need for stronger community engagement and the implementation of tailored community-based awareness raising and screening programs that engage trusted community leaders and utilize outreach strategies targeting high-risk populations, such as people who inject drugs (PWID) and pregnant women [43].

We found that limited availability of diagnostic testing for hepatitis B and C at the primary care level was another important barrier to care. Although screening tests (i.e. hepatitis B surface antigen and hepatitis C antibody) were available at some primary care facilities, HCV RNA and HBV DNA testing were, with a few exceptions, only available at the provincial hospital or end referral facilities. Accessibility of viral load testing is a well-recognized challenge to the scale-up of hepatitis diagnosis and treatment [34, 44, 45]. To help address this, WHO, in their latest guidelines on HBV infection, updated treatment eligibility criteria with only one of four options requiring access to HBV DNA testing [34]. Some countries have started to integrate HBV DNA and HCV RNA testing platforms with other disease programs, such as HIV or tuberculosis, to improve efficiency and reduce costs [46]. Decentralization of PCR platforms to the primary care level may not be feasible or cost-effective in all settings so alternatives such as point-of-care tests, dried-blood spots, or mechanisms for plasma specimen transport to more centralized laboratories are needed. In addition, several alternative methods have recently shown promise in identifying treatment-eligible individuals in settings where HBV DNA testing is unavailable [47, 48].

We also found a lack of availability of antiviral medications, particularly for HCV infection, at the primary care level. Globally the cost of HCV and HBV treatment has declined significantly [45, 49] however this lower cost has not always translated into better accessibility especially at the primary care level [46]. Challenges such as fragmented procurement systems, weak inventory and forecasting systems, and inadequate distribution networks contribute to this discrepancy [46]. Our data is consistent with that reported by the WHO in its latest global hepatitis report. According to the WHO survey of 38 focus countries globally, 94% have included TDF in their national viral hepatitis guidelines, 79% have included it in national essential medicines lists, but only 45% have TDF available for use in primary healthcare [46]. Similarly, for HCV 88% of the countries have included SOF and DAC in their national viral hepatitis guidelines, 57% have included it in national essential medicines lists but only 30% have SOF and DAC available for use in primary healthcare [46].

Even when diagnostics and treatment are available at the primary care level, affordability remains a significant barrier in both countries. Importantly, our data demonstrates that social health insurance coverage for hepatitis services is not available at the primary care level. In Viet Nam, health insurance coverage in Thai Binh province reached 92% of the population in 2023. While Viet Nam’s SHI policy provides coverage for outpatient hepatitis treatment (up to 80% for HBV and 50% for HCV medications) [9–11], our findings demonstrate this has not translated to practice, meaning that many patients have to pay significant out-of-pocket costs for hepatitis care [50, 51]. Potential reasons for this gap include a lack of health insurance accreditation at certain facilities and patients being ineligible for reimbursement because of self-referral to a higher-level facility. In the Philippines, there is no general outpatient PhilHealth coverage for hepatitis with the exception of specific populations [9, 52]. Hepatitis care can sometimes be covered by agencies such as the DOH and local government units. While these agencies provide diagnostic testing kits and medications, their limited availability can be a source of frustration for both the healthcare workers and patients. Furthermore, the fragmented nature of health coverage, with services being funded by multiple agencies, contribute to inconsistent coverage for hepatitis services [52]. Overall, the lack of insurance coverage at the primary care level is a major deterrent to decentralizing hepatitis services for both facility managers and the community.

Referral pathways between primary care and specialist services appear to be functioning relatively well in the Philippines and Viet Nam. However, the absence of strong back-referral processes may result in gaps in patient follow-up and limited communication regarding ongoing treatment at the primary care level. Strengthening these back-referral mechanisms through enhanced coordination, standardized protocols, and electronic record-keeping would enable primary care providers to stay informed and play a more active role in ongoing patient care. Implementing well-coordinated referral and back-referral systems requires strategies tailored to local health system structures, workforce capacities, and technological resources [53]. Provider training in patient handover processes and in accessible communication tools, such as secure messaging applications, to bridge gaps in continuity of care. By adapting these best practices [53], countries can improve treatment outcomes, enhance patient management, and reduce healthcare fragmentation, ensuring patients receive consistent follow-up and primary care teams remain engaged in long-term management.

Addressing the gaps identified in this study is essential to achieving the hepatitis elimination targets set for 2030. Effective decentralization of hepatitis services will require significant efforts to ensure that primary care facilities are ready to deliver high-quality people-centered care for people living with viral hepatitis. Such efforts should prioritize the lived experiences of people with hepatitis and involve them in the design of health services [54]. Both Viet Nam and the Philippines have national action plans and relatively robust policy frameworks for hepatitis elimination which include decentralization of hepatitis care to the primary healthcare level [9, 52]. However, translating national guidelines and action plans into effective service delivery at the primary care level requires reforms at the subnational level. This includes efforts to increase health insurance coverage for hepatitis care while also accrediting primary care facilities to be eligible to provide care under SHI. Improving access to confirmatory diagnostics and antiviral treatment can be achieved through focused procurement and supply chain management. Targeted interventions such as enhanced healthcare worker capacity building, improved diagnostic infrastructure, stronger community engagement, tailored community-based screening, and more robust referral networks are needed.

Our study has several limitations. First, it focused geographically on Thai Binh province in Viet Nam and Central Luzon in the Philippines, which limits the generalizability of the findings. With only 18 health facilities included, the conclusions may not apply broadly to other settings. Second, while the HFA tool was adapted from the WHO SARA instrument, our study used a self-administered survey rather than the direct observation methodology recommended by WHO. This may introduce response bias, particularly in the reporting of service availability or staff capacity. To mitigate this limitation, we incorporated follow-up site visits at each facility to validate responses through brief interviews and observation, thereby improving the reliability of the data. Additionally, the self-reported nature of data from healthcare providers may have introduced biases in perceptions regarding service availability, affordability, and healthcare provider capabilities. Finally, while the study addresses important supply-side factors (e.g., service availability, healthcare worker capacity, and medication supply), it provides limited insights into demand-side barriers, such as patients’ experiences, social determinants of health, or stigma that might influence access to care [51].

For future studies, expansion of the geographic scope and facility coverage to a national level should improve the external validity of the findings across diverse health system contexts. Multisite studies encompassing additional provinces and urban-rural gradients in both Viet Nam and the Philippines would allow comparative analyses and strengthen generalizability. To enhance the accuracy of the measurement, subsequent research could employ hybrid data collection designs that combine WHO SARA-based instruments or checklists with structured provider interviews or digital audits, reducing reliance on self-reported data and minimizing response biases. Incorporating patient-level perspectives through mixed method approaches, such as household surveys, patient journey mapping, and focus group discussions, would provide deeper insights into demand-side barriers and behavioral determinants influencing access to care. Lastly, conducting longitudinal research on the evaluation of how health facility readiness evolves over time, particularly after targeted interventions or policy reforms, could further illuminate causal pathways between system capacity and service delivery outcomes. Such evidence would not only refine national hepatitis program strategies but also inform scalable models for strengthening primary care readiness across comparable low to middle income country settings.

## Conclusion

This study identified critical gaps in the readiness of primary healthcare systems in both Viet Nam and the Philippines to provide comprehensive hepatitis services. While efforts toward decentralization and integration of hepatitis B and C care into the primary healthcare level are underway, significant challenges remain. These include limited availability of essential commodities and services, inadequate health workforce training, insufficient community awareness and engagement, and weak financial support mechanisms. Addressing these gaps will require coordinated efforts from national and subnational stakeholders. Notably, the lack of coverage by social health insurance for hepatitis services at the primary care level presents a major barrier to care that must be addressed if Viet Nam and the Philippines are to meet the 2030 hepatitis elimination targets.

## Electronic supplementary material

Below is the link to the electronic supplementary material.

Supplementary Material 1

Supplementary Material 2

## Data Availability

The datasets used and/or analysed during the current study are available from the corresponding author on reasonable request.
