# Supplementary material for: Healthcare system readiness to manage viral hepatitis in Viet Nam and the Philippines: results of a brief health facility assessment
Source: BMC Health Serv Res. 2026 Feb 2;26:326. doi: 10.1186/s12913-026-14088-y (PMC12954926; doi:10.1186/s12913-026-14088-y)
Supplement: Supplementary file 1 — Supplementary Material 1 [file 12913_2026_14088_MOESM1_ESM.pdf]

Health Facility Assessment: Focus Group Discussion

Facilitator/s: ______________________

No.	Key contact person/s (names)	Position/Title	Health Facility	Contact number/s	Email Address/s
1					
2					
3					
4					
5					
6					
7					
8					
9					
10					
**Date and Time of Interview**					

Introduction

Good day! Thank you for agreeing to join us today and to be a part of the study on understanding the current care for patients with chronic viral hepatitis in the [**country**]. My name is [**facilitator name**], as you might know, I am a part of a research team and will be facilitating the focus group discussion today.

As healthcare workers with experience delivering care within the facilities that we are working with, we are interested in sharing some of the things that we have learned from the surveys of the facilities and gaining your perspectives. This will help us to have a deeper understanding about what some of the challenges and opportunities are for improving care.

You have all already signed the consent form, thank you. Before we begin, I just want to emphasize that all the information you will share today will be kept confidential and not be attributable to you (beyond, of course, the people in attendance today), and will have no consequences for you professionally, so you can feel free to answer as honestly as you would like

<table>
<tr><th>QUESTIONS</th><th>PROBES</th></tr>
<tr><td colspan="2">Introduction and Hepatitis Knowledge</td></tr>
<tr><td>1. How well do you think hepatitis is managed in the country? In your health facility? What is going well and not so well?</td><td>What is going well/done according to policy/protocols? And what is not?</td></tr>
<tr><td>Screening and Diagnosis</td><td></td></tr>
<tr><td>2. Through what method and in what context are most of your patients (if any) diagnosed with hepatitis?</td><td>ie. screening programs, opportunistic testing, presenting with signs and symptoms of hepatitis, etc.</td></tr>
<tr><td>3. What are some of the challenges to screening and diagnosis of hepatitis in your country/health facility?</td><td>Refer to relevant findings from HFA (e.g., availability of tests, etc.)</td></tr>
<tr><td colspan="2">Specific issues identified:
a. Availability of screening tests (ie. inconsistent supply - esp. for HCV; inconsistent outpatient coverage [prenatal screening, blood donors, and HIV patients are screened freely])
b. Coverage of diagnostic tests for further evaluation (including viral load testing and other diagnostics [hepatitis profile, imaging, etc.])</td></tr>
<tr><td>Management and Adherence</td><td></td></tr>
<tr><td>4. What are the main steps you (would) undertake in managing a patient with hepatitis after diagnosis?</td><td></td></tr>
<tr><td>5. What are some of the challenges to initiating and continuing the management of patients with hepatitis in your country/health facility?</td><td>Refer to relevant findings from HFA (e.g. medication availability, etc.)

Probe about availability of resources (medications, tools), coordination of care (referral and back-referral systems), healthcare worker factors (capability/capacity)</td></tr>
<tr><td colspan="2">Specific issues identified:
a. Availability of medications (ie. inconsistent supply, limited window - near expiring medications)
b. Lack of training in some health facilities for the management of HCV and HBV
c. Diversity of roles of healthcare workers in health facilities (not only focused on Hepatitis cases but may assist in other areas of the hospital [for ERFs] or other health facilities [for SATFs])</td></tr>
<tr><td>Perspective of patient barriers and enablers</td><td></td></tr>
<tr><td>6. What do you think are some of the most important enablers for patients obtaining care for hepatitis?</td><td rowspan="2">Probe for individual, social, community, and health system factors along the cascade of care (screening/diagnosis, linkage to care, initiation of treatment, adherence); insurance coverage

ie. Finance capacity, knowledge, information, availability of healthcare services, support of patient relatives, etc.</td></tr>
<tr><td>7. What do you think are some of the most important challenges for patients obtaining care for hepatitis?</td></tr>
<tr><td>8. Are there things about the current program for HBV and HCV that are very helpful for patients obtaining care for hepatitis and should be retained or enhanced?</td><td></td></tr>
<tr><td>9. Are there particular groups of patients that need a different</td><td>Probe about key population groups here, capacity for</td></tr>
</table>

approach? Such as more support?	individualized person-centered care
10. What do you think about the patients' experiences in receiving care for HBV and HCV within the current healthcare system?	Is it easy or difficult to navigate?<br>What do the patients think about the quality of care?<br>How well do they interact with healthcare providers?
**Conclusions and ways forward**	
11. If there was one thing that could be done to improve outcomes and care for patients with hepatitis, what would it be?	Any other suggestions to overcoming the barriers for patient care for hepatitis?
12. Is there anything else you would like to share with us today?	

Conclusion

Thank you very much for everyone's participation. Your responses will help us to better understand the care that patients with hepatitis receive and how it might be improved.

We will be sharing the results of the facility assessment and some of the themes from our discussion today with the health facilities and community members next month, and we hope you can join us.

We will also be using this to move to a design and implementation phase of interventions to improve care for patients with hepatitis and may be in touch again related to activities to support that.
