# Supplementary material for: Healthcare system readiness to manage viral hepatitis in Viet Nam and the Philippines: results of a brief health facility assessment
Source: BMC Health Serv Res. 2026 Feb 2;26:326. doi: 10.1186/s12913-026-14088-y (PMC12954926; doi:10.1186/s12913-026-14088-y)
Supplement: Supplementary file 2 — Supplementary Material 2 [file 12913_2026_14088_MOESM2_ESM.pdf]

Health Facility Assessment: Health Facilities

General Facility Data

<table>
<tr><td>Facility Number</td><td colspan="3"></td></tr>
<tr><td>Facility Name</td><td colspan="3"></td></tr>
<tr><td rowspan="2">Facility Category</td><td>The Philippines
[] Screening, Assessment, and Treatment Facility (SATF)
[] Barangay Health Center
[] RHU
[] CHO
[] BEmONC Facility
[] Social Hygiene Clinic</td><td>[] Screening, Assessment, and Treatment Facility (SATF)
[] HIV Treatment Hub
[] Primary HIV Care Clinic
[] Hospital (Infirmary)
[] Hospital (Level 1)
[] Hospital (Level 2)
[] Others: ____________</td><td>[] End Referral Facility (ERF)
[] Level 3 Hospital</td></tr>
<tr><td colspan="3">Vietnam
[] Provincial hospital
[] District hospital/health center
[] Commune health station</td></tr>
<tr><td>Facility Type</td><td colspan="3">[] Public [] Private</td></tr>
<tr><td>Complete Address of Facility</td><td colspan="3"></td></tr>
<tr><td>Head of Facility</td><td colspan="3"></td></tr>
<tr><td>Key contact person/s</td><td>1.</td><td>2.</td><td>3.</td></tr>
<tr><td>Contact number/s</td><td></td><td></td><td></td></tr>
<tr><td>Email Address/s</td><td></td><td></td><td></td></tr>
<tr><td>Facility Operations (Day and Time)</td><td>[] Monday ____ HH:MM - ____ HH:MM
[] Tuesday ____ HH:MM - ____ HH:MM
[] Wednesday ____ HH:MM - ____ HH:MM</td><td>[] Thursday ____ HH:MM - ____ HH:MM
[] Friday ____ HH:MM - ____ HH:MM</td><td>[] Saturday ____ HH:MM - ____ HH:MM
[] Sunday ____ HH:MM - ____ HH:MM</td></tr>
<tr><td>Date and Time of Interview</td><td colspan="3"></td></tr>
</table>

<u>Introduction</u>

Good day! Thank you for your participation in the survey. The survey includes questions related to the general and specific service readiness and availability of Hepatitis B and C healthcare services in your facility. Please answer each of the following questions with the best possible answer.
The survey covers many aspects of Hepatitis B and C healthcare services, therefore you can work as a group to answer the questions.
*Note: You may place **N/A** if not applicable or not available in your facility*

<u>General Service Availability</u>

I. Health Facility Service Availability

1. The selected facility has the following patient services:	[] Outpatient consultations<br>[] In patient rooms, [] Private [] Charity<br>[] Emergency room<br>[] Pharmacy<br>[] Diagnostics<br>[] Laboratory<br>[] Imaging, [] CT [] MRI [] Xray [] UTZ [] Others: _____	[] Rooms for surgical procedures<br>[] Operating room<br>[] Birthing rooms<br>[] Recovery rooms<br>[] Others: _______
2. Total number of in-patient beds		

II. Patient Census (Outpatient)

1. The selected facility has a recent record/report on the outpatient census (2022)	[] Yes [] No
2. Total number of the total outpatient population in the selected facility based on the latest census (2022)	
3. Total number of prenatal consults at the selected facility (2022)	

III. Health workforce

Number of healthcare staff in the selected facility (2022):	
1. Physician/s (General Physicians)	____
2. Physician/s (Specialists)	____
2a. Gastroenterologists	____
2b. Hepatologists	____
2c. IDS	____
2d. Radiologists	____
2e. Others: (Type and Number)	[] Type:________ Amount: ________<br>[] Type:________ Amount: ________

3. Nurse/s	____
4. Midwive/s	____
5. Medical Technologists/s	____
6. Barangay Health Worker/s (BHW)	____
7. Others: (Name and Number)	[] Type:________ Amount: ________<br>[] Type:________ Amount: ________<br>[] Type:________ Amount: ________

Specific Service Availability for Hepatitis

I. Screening

**HEPATITIS B**	
The selected facility has a recent record/report on the number of screening done for hepatitis B on routine visits (2022)?	[] Yes [] No
Total population of patients screened for hepatitis B on routine visits	____
Total population of patients who were found reactive for hepatitis B on routine visits	____
**HEPATITIS C**	
The selected facility has a recent record/report on the number of screening done for hepatitis C on routine visits (2022)?	[] Yes [] No
Total population of patients screened for hepatitis C on routine visits	____
Total population of patients who were found reactive for hepatitis C on routine visits	____

II. Management

**HEPATITIS B** (2022)	
The selected facility has a recent record/report on the number of patients undergoing **Hepatitis B management**	[] Yes [] No
Total population of patients who are hepatitis B reactive and is eligible for treatment	
Total population of patients who are hepatitis B reactive, eligible for treatment, and are undergoing treatment	
Total population of patients who were found reactive for Hepatitis B, is **not** eligible for treatment, and is undergoing monitoring	
The total number of chronic Hepatitis B cases who were lost to follow up (Patients who did not return for follow up, did not complete evaluation for eligibility of treatment, and/or discontinued medication without follow up)	
The total number of chronic Hepatitis B cases who developed complications (hepatocellular carcinoma, cirrhosis, etc.) of the selected facility	
**HEPATITIS C** (2022)	
The selected facility has a recent record/report on the number of patients undergoing **Hepatitis C management**	[] Yes [] No
Total population of patients who are hepatitis C reactive and are undergoing treatment	
Total population of patients who were found reactive for Hepatitis C and completed treatment	____
Total population of patients who were found reactive for Hepatitis C and completed treatment that were classified as **sustained virological response (SVR)**	
Total population of patients who were found reactive for Hepatitis C and completed treatment that were classified as **treatment failure**	
The total number of chronic Hepatitis C cases who were lost to follow up (Patients who did not return for follow up, did not complete evaluation for eligibility of treatment, and/or discontinued medication without follow up)	
The total number of chronic Hepatitis C cases who developed complications (hepatocellular carcinoma, cirrhosis, etc.) of the selected facility	

Adequate Service Specific Readiness and Capacity for Hepatitis

I. Primary Health Care Vaccination Program

1. The facility provides hepatitis B vaccination services	[] Yes [] No
1a. Among what conditions are the hepatitis B vaccinations provided in?	*Conditions that Hepatitis B vaccinations are provided in:*<br>*[] Infant*<br>*[] Catch up (Pediatric)*<br>*[] Catch up (Adult)*<br>*[] Adult (Pregnant)*<br>*[] Adult (Non-pregnant)*<br>*[] Others: _____________*
2. How many doses of Hepatitis B vaccines have been provided in the past year (2022)?	
3. Number of identified patients for vaccination of Hepatitis B in the past year (2022)?	
4. Number of fully vaccinated patients for hepatitis B in the past year (2022)?	

II. Information, Education, and Communication (IEC) Campaign

1. Has the health facility provided IEC for hepatitis healthcare services in the past year (2022)?	[] Yes [] No
2. What types of IEC campaigns have been implemented in the past year (2022)?	*List of types of IECs implemented:*<br>-<br>-
3. Total population who accessed the IEC campaign in the past year (2022)?	

III. Safe Blood Transfusion

1. Does the facility implement safe blood transfusion?	[] Yes [] No
2. Does the facility have a written health facility specific protocol/policy based on the national safe blood transfusions guidelines?	[] Yes [] No

IV. Prevention of Hepatitis B and C virus infection at health facilities

1. Does the facility implement infection control programs specific for Hepatitis B and C in the past year (2022)?	[] Yes [] No
1a. If yes, what are the activities of the program?	*List of activities for HBV and HCV Infection prevention and control:*<br>-<br>-

V. Management

A. Screening Program

1. Does the health facility provide **rapid diagnostic testing screening** for Hepatitis B and C?	[] Yes [] No	
**1a. Hepatitis B and C**	**HBV**	**HCV**
1ai. Active surveillance screening program?	[] Yes, Name/s of program:<br>-<br>-<br>[] No	[] Yes, Name/s of program:<br>-<br>-<br>[] No
1aii. Opportunistic screening (offered screening of HBV/HCV during routine visits)?	[] Yes, Type/s of eligible population:<br>-<br>-<br>[] No	[] Yes, Type/s of eligible population:<br>-<br>-<br>[] No
2. **Rapid diagnostic test use**	[] Yes [] No	[] Yes [] No
2a. Number of rapid diagnostic tests used in the past year (2022) for screening?	________________	________________
3. Do the patients receive **counseling** with the use of the rapid diagnostic tests?		
3a. Pre-test counseling	[] Yes [] No	[] Yes [] No
3b. Post-test counseling	[] Yes [] No	[] Yes [] No
3c. If yes, who conducts the counseling (check all that applies)?	[] BHW<br>[] Nurse<br>[] Midwife<br>[] Physician	[] BHW<br>[] Nurse<br>[] Midwife<br>[] Physician

	[] Others: _______	[] Others: _______
4. Does the facility provide pre-employment hepatitis screening for companies/organizations?	[] Yes [] No	[] Yes [] No
4a. If yes, how many organizations/companies have requested for pre-employment hepatitis screening in the most past year (2022)?	________	

B. Diagnostics

**LABORATORY EXAMINATIONS**			
**1. HBsAg (rapid diagnostic test or laboratory based immunoassay)**	[] Yes, available at the current facility<br>[] Yes, at a nearby referral facility<br>[] No	**8. HIV rapid diagnostic test**	[] Yes, available at the current facility<br>[] Yes, at a nearby referral facility<br>[] No
**2. HBV DNA (quantitative)**	[] Yes, available at the current facility<br>[] Yes, at a nearby referral facility<br>[] No	**9. ALT, AST**	[] Yes, available at the current facility<br>[] Yes, at a nearby referral facility<br>[] No
**3. HBeAg**	[] Yes, available at the current facility<br>[] Yes, at a nearby referral facility<br>[] No	**10. Platelet count**	[] Yes, available at the current facility<br>[] Yes, at a nearby referral facility<br>[] No
**4. Anti-HBs**	[] Yes, available at the current facility<br>[] Yes, at a nearby referral facility<br>[] No	**11. Creatinine**	[] Yes, available at the current facility<br>[] Yes, at a nearby referral facility<br>[] No
**5. Anti-HBe**	[] Yes, available at the current facility<br>[] Yes, at a nearby referral facility<br>[] No	**12. Alpha-fetoprotein (AFP)**	[] Yes, available at the current facility<br>[] Yes, at a nearby referral facility<br>[] No
**6. Anti HCV rapid diagnostic test**	[] Yes, available at the current facility<br>[] Yes, at a nearby referral facility<br>[] No	**13. Other Hepatitis B or C related tests, specify:** _____________	[] Yes, available at the current facility<br>[] Yes, at a nearby referral facility<br>[] No
**7. HCV RNA (quantitative)**	[] Yes, available at the current facility<br>[] Yes, at a nearby referral facility<br>[] No		

IMAGING STUDIES			
**1. Ultrasonography**	[] Yes, available at the current facility<br>[] Yes, at a nearby referral facility<br>[] No	**3. Magnetic Resonance Imaging (MRI)**	[] Yes, available at the current facility<br>[] Yes, at a nearby referral facility<br>[] No
**2. Computed Tomography Scans (CT-scans)**	[] Yes, available at the current facility<br>[] Yes, at a nearby referral facility<br>[] No		
**CONFIRMATORY TEST**			
**1. Liver biopsy (at the ERF)**		[] Yes, available at the current facility<br>[] Yes, at a nearby referral facility<br>[] No	

**SUPPLIES**	
Available Antiviral Medications:	
1. Nucleos(t)ide analogues	
1a. Tenofovir disoproxil fumarate (TDP)	[] Yes [] No
1b. Tenofovir alafenamide (TAF)	[] Yes [] No
1c. Entecavir (ETV)	[] Yes [] No
1d. Other Nucleos(t)ide analogues	[] Yes [] No<br>If Yes, Specify: ____________
2. Sofosbuvir	[] Yes [] No
3. Sofosbuvir/Velpatasvir	[] Yes [] No
4. Daclatasvir	[] Yes [] No
5. Others	[] Name: ____________<br>[] Name: ____________<br>[] Name: ____________

Available Vaccines (Immunization):	
1. Hepatitis B Vaccine (Infant)	[] Yes [] No
2. Hepatitis B Vaccine (Adult)	[] Yes [] No

VI. Health Human Resources

A. Census

Number of trained healthcare staff in Hepatitis B and C management:	**HBV**	**HCV**
1. Physician/s (General Physicians)		
2. Physician/s (Specialists)		
2a. Gastroenterologist		
2b. Hepatologist		
2c. IDS		
2d. Others: (Type and number)	[] Type:_________ Amount: ___<br>[] Type:_________ Amount: ___<br>[] Type:_________ Amount: ___	[] Type:_________ Amount: ___<br>[] Type:_________ Amount: ___<br>[] Type:_________ Amount: ___
3. Nurse/s		
Number of medical technologists trained in Hepatitis B and C laboratory testing services		
Number of midwives or other health personnel oriented in health communication for Hepatitis B and C		

B. Training

Trained healthcare staff on (**Hepatitis B and C** management):	**HBV**	**HCV**
1. Did the health facility conduct training for staff or send staff to train on courses on Hepatitis management in the past year (2022)?	[] Yes [] No	[] Yes [] No
1a. If yes, when was the most recent training conducted?	[Date]: __ /__/__ (MM/DD/YY)	[Date]: __ /__/__ (MM/DD/YY)
1b. If yes, how many health care staff participated in these training sessions?	[] General Physicians (No. ____)	[] General Physicians (No. ____)

	[] Specialists (No. ___)<br>[] Nurses (No. ___)<br>[] Midwives (No. ___)<br>[] Medical Technologists (No. ___)<br>[] Others<br>[] __________ (No. ___)<br>[] __________ (No. ___)<br>[] __________ (No. ___)	[] Specialists (No. ___)<br>[] Nurses (No. ___)<br>[] Midwives (No. ___)<br>[] Medical Technologists (No. ___)<br>[] Others<br>[] __________ (No. ___)<br>[] __________ (No. ___)<br>[] __________ (No. ___)
1c. What topics were discussed in the most recent training session?	[] Identification of risk factors<br>[] Management<br>[] Criteria in the initiation of treatment (and contraindications)<br>[] Complications<br>[] Patient education<br>[] Counseling<br>[] Monitoring<br>[] Criteria in discontinuation of treatment<br>[] Others: _______	[] Identification of risk factors<br>[] Management<br>[] Criteria in the initiation of treatment (and contraindications)<br>[] Complications<br>[] Patient education<br>[] Counseling<br>[] Monitoring<br>[] Criteria in discontinuation of treatment<br>[] Others: _______

VII. Finances

	**HBV**	**HCV**
1. Are **screening tests** covered?	[] Yes [] No	[] Yes [] No
	The Philippines<br>[] DOH [] LGU [] PhilHealth<br>[] HMO (specify): ________<br>[] Others: ______	The Philippines<br>[] DOH [] LGU [] PhilHealth<br>[] HMO (specify): ________<br>[] Others: ______
1a. If yes, covered by who?	Vietnam<br>[] Social health insurance<br>[] Others (specify): ______	Vietnam<br>[] Social health insurance<br>[] Others (specify): ______

1b. If yes, in which medical condition/s?	*Conditions eligible for coverage*:<br>-<br>-	*Conditions eligible for coverage*:<br>-<br>-
1c. If yes and by PhilHealth or HMO (the Philippines) / Social health insurance (Vietnam), what is the percent of cost covered?	The Philippines<br>[] PhilHealth (___ %) [] HMO (___ %)	The Philippines<br>[] PhilHealth (___ %) [] HMO (___ %)
	Vietnam<br>[] Social health insurance (___ %)	Vietnam<br>[] Social health insurance (___ %)
2. Is **viral load testing** covered?	[] Yes [] No	[] Yes [] No
2a. If yes, covered by who?	The Philippines<br>[] DOH [] LGU [] PhilHealth<br>[] HMO (specify): ________<br>[] Others: ______	The Philippines<br>[] DOH [] LGU [] PhilHealth<br>[] HMO (specify): ________<br>[] Others: ______
	Vietnam<br>[] Social health insurance<br>[] Others (specify): ______	Vietnam<br>[] Social health insurance<br>[] Others (specify): ______
2b. If yes, in which medical condition/s?	*Conditions eligible for coverage*:<br>-<br>-	*Conditions eligible for coverage*:<br>-<br>-
2c. If yes and by PhilHealth or HMO / Social health insurance (Vietnam), what is the percent of cost covered?	The Philippines<br>[] PhilHealth (___ %) [] HMO (___ %)	The Philippines<br>[] PhilHealth (___ %) [] HMO (___ %)
	Vietnam<br>[] Social health insurance (___ %)	Vietnam<br>[] Social health insurance (___ %)
3. Are **medications** covered?	[] Yes [] No	[] Yes [] No
3a. If yes, what medications are covered?	*List of medications:*<br>-<br>-	*List of medications:*<br>-<br>-
3b. If yes, covered by who?	The Philippines<br>[] DOH [] LGU [] PhilHealth<br>[] HMO (specify): ________<br>[] Others: ______	The Philippines<br>[] DOH [] LGU [] PhilHealth<br>[] HMO (specify): ________<br>[] Others: ______

	Vietnam<br>[] Social health insurance<br>[] Others (specify): ______	Vietnam<br>[] Social health insurance<br>[] Others (specify): ______
3c. If yes, in which medical condition/s?	*Conditions eligible for coverage*:<br>-<br>-	*Conditions eligible for coverage*:<br>-<br>-
3d. If yes and by PhilHealth or HMO / Social health insurance (Vietnam), what is the percent of cost covered?	The Philippines<br>[] PhilHealth (___ %) [] HMO (___ %)	The Philippines<br>[] PhilHealth (___ %) [] HMO (___ %)
	Vietnam<br>[] Social health insurance (___ %)	Vietnam<br>[] Social health insurance (___ %)
4. Is **consultation** covered?	[] Yes [] No	[] Yes [] No
4a. If yes, covered by who?	The Philippines<br>[] DOH [] LGU [] PhilHealth<br>[] HMO (specify): ________<br>[] Others: ______	The Philippines<br>[] DOH [] LGU [] PhilHealth<br>[] HMO (specify): ________<br>[] Others: ______
	Vietnam<br>[] Social health insurance<br>[] Others (specify): ______	Vietnam<br>[] Social health insurance<br>[] Others (specify): ______
4b. If yes, in which medical condition/s?	*Conditions eligible for coverage*:<br>-<br>-	*Conditions eligible for coverage*:<br>-<br>-
4c. If yes and by PhilHealth or HMO / Social health insurance (Vietnam), what is the percent of cost covered?	The Philippines<br>[] PhilHealth (___ %) [] HMO (___ %)	The Philippines<br>[] PhilHealth (___ %) [] HMO (___ %)
	Vietnam<br>[] Social health insurance (___ %)	Vietnam<br>[] Social health insurance (___ %)

- THE END -
